# The role of cannabis and salience alterations in determining the severity of psychotic symptoms: a multi-centric, cross-sectional study on adolescent and adult cohorts

**DOI:** 10.1192/j.eurpsy.2024.1538

**Published:** 2024-08-27

**Authors:** O. Baccaredda Boy, G. P. Merola, A. Patti, I. Fascina, B. Bozza, D. Flaccomio, M. Faldi, G. Pitt, I. Noschese, L. Papini, D. Brugnolo, C. Ricci, V. Pecoraro, A. Ballerini, V. Ricca, F. Mauceri, G. Peroni, S. Tavano, T. Pisano, F. De Cesaris, S. Gori, D. Cohen

**Affiliations:** ^1^University of Florence, Florence, Italy; ^2^Department of Pitié Salpêtrière, University of Sorbonne, Paris, France

## Abstract

**Introduction:**

The aim of this project is to study to which extent salience alterations influence the severity of psychotic symptoms. However, rather than studying them individually, we decided to focus on their interplay with two additional variables, that is: observing their effect in a vulnerability phase (adolescence) and with another added, well-recognized risk factor (cannabis use).

The reason for this study design lies in the fact that, in our opinion, it is fundamental to observe the trajectory of psychotic symptoms over a continuum; however, rather than adopting a longitudinal approach, we decided to structure it as a cross-sectional study confronting patients from two age brackets - adolescence and adulthood.

**Objectives:**

The primary purpose of this study was to assess a difference between THC-abusing and non-abusing patients in adolescent and adult cohorts, using the Italian version of the psychometric scale “Aberrant Salience Inventory” (ASI), and the possible correlation with more severe psychotic symptoms. The employment of several different psychometric scales and the inclusion of a variegated cohort allowed to pursue multiple secondary objectives.

**Methods:**

We recruited 192 patients, subsequently divided into six subgroups based on age and department of recruitment (whether adolescent or adult psychiatric or neurologic units - the latter serving as controls). Each individual was administered a set of questionnaires and a socio-demographic survey; the set included: Aberrant Salience Inventory (ASI), Community Assessment of Psychic Experiences (CAPE), Positive and Negative Syndrome Scale (PANSS), Montgomery-Asberg Depression Rating Scale (MADRS), Mania Rating Scale (MRS), Hamilton Anxiety Scale (HAM-A), Association for Methodology and Documentation in Psychiatry (AMDP) and Cannabis Experience Questionnaire (CEQ).

**Results:**

The data analysis showed statistically significant (p<0.05) differences between adolescents and adults with psychotic symptoms in all of the three scales of PANSS and in MADRS. These two groups were homogenous for both cannabis use and ASI score. The intra-group comparison (either adolescent or adult) showed a hierarchical pattern in the scores of psychometric scales according to the diagnostic subgroup of allocation: patients with psychotic symptoms showed an higher level of psychopathology in all measures when compared to patients from the psychiatric unit without psychotic symptoms, which in turn scored higher than the patients from the neurologic unit.

**Image:**

**Figure:**
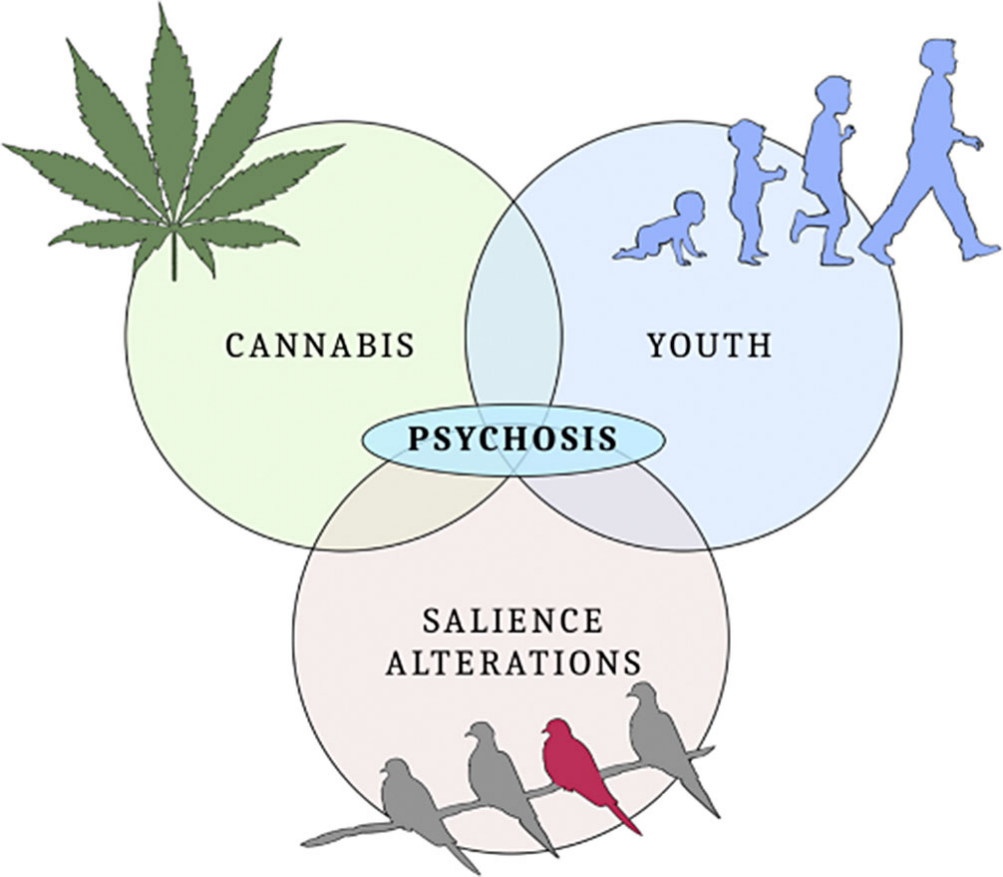

**Conclusions:**

The results of the present study may suggest that when salience alterations occur in adolescents with cannabis exposure, we might observe worsened positive and negative psychotic symptoms; their influence might be relevant also in other domains, especially regarding the depressive and anxiety spectrums.

**Disclosure of Interest:**

None Declared

